# Lethal and Sublethal Effects of Methyl Benzoate on the Predatory Bug *Nesidiocoris tenuis*

**DOI:** 10.3390/insects11060377

**Published:** 2020-06-18

**Authors:** Md Munir Mostafiz, Errol Hassan, Jae-Kyoung Shim, Kyeong-Yeoll Lee

**Affiliations:** 1 Division of Applied Biosciences, College of Agriculture and Life Sciences, Kyungpook National University, Daegu 41566, Korea; munirmostafiz12@gmail.com; 2 School of Agriculture and Food Sciences, The University of Queensland Gatton, Queensland 4343, Australia; e.hassan@uq.edu.au; 3 Institute of Agricultural Science and Technology, Kyungpook National University, Daegu 41566, Korea; astelia@naver.com; 4 Sustainable Agriculture Research Center, Kyungpook National University, Gunwi 39061, Korea

**Keywords:** biopesticides, natural compound, natural enemy, *Nesidiocoris tenuis*, sustainable agriculture

## Abstract

Benzoates (naturally occurring plant toxins) produce pesticidal effects on various pest insects and mites, but their effects on non-target insects are poorly understood. In this study, we evaluate the lethal and sublethal toxicity of methyl benzoate (MB) to adults of the generalist predatory bug *Nesidiocoris tenuis* (Reuter) (Hemiptera: Miridae). To assess lethal effects, *N. tenuis* was exposed to plant surfaces treated with 0.25%, 0.5% and 1% MB, as well as negative and positive controls (water and the neonicotinoid acetamiprid, respectively). Exposure to 1% MB resulted in the highest corrected mortality of 17.8% and 13.3% under laboratory and greenhouse conditions, respectively. Thus, 1% MB can be classified as harmless to *N. tenuis* according to the International Organization for Biological Control rating scheme. At the sublethal level, MB exposure did not significantly affect the consumption of eggs of the whitefly *Bemisia tabaci* by *N. tenuis* relative to negative control feeding rates. In contrast, acetamiprid at the manufacturer’s recommended concentration reduced *N. tenuis* feeding activity by 45.4%. Furthermore, in a Y-tube olfactometer assay, there were no significant differences between the olfactory responses of *N. tenuis* to MB concentrations and the negative control (water). This study therefore suggests that MB could be used safely for pest control in combination with *N. tenuis*.

## 1. Introduction

Integrated pest management (IPM) approaches, in which both biological and chemical control agents are applied against pests, are favored over pesticide-only approaches in sustainable production systems [1,2]. However, the use of broad-spectrum chemicals in pest management can have negative effects on a target pest’s natural enemies and may impact the efficacy of these enemies as biological control agents [3,4,5]. Therefore, further research into the adverse effects of pesticides on natural enemies is crucial for maintaining and improving the effectiveness of biological control systems and for evaluating the suitability of pesticides in IPM programs [1]. Such research should assess the lethal and sublethal effects of chemical pesticides on natural enemies.

Lethal effects include any acute toxicity effects (i.e., mortality) of pesticides, whereas sublethal effects can be behavioral and/or physiological effects on individuals, including changes to their development, longevity, and/or reproduction [1,6]. For the natural enemies of pest species, the risk assessment of pesticides is generally based on both lethal and sublethal effects. Traditionally, evaluation of pesticides for both registration purposes and compliance with IPM systems begins with an analysis of their acute toxicity, which provides fundamental data on the potential threat they pose to natural enemies [7].

It is also important to consider various methods when evaluating the toxicity of pesticides to natural enemies, as well as the many potential routes of toxicant penetration into the animals’ bodies. Testing conditions could include any of the following: topical application used to replicate direct exposure of the natural enemies to spray droplets [8]; residual toxicity studies, in which chemicals applied to plant surfaces or inert substrates replicate the interaction of natural enemies with crop residues [9]; treated plants, in which pesticides in plant tissue contaminate plant resources, such as nectar or plant sap, that may be essential for the nutrition of natural enemies [10]; and treated prey or hosts, since toxicants can transfer topically or by ingestion during predation or parasitism [11]. The importance of these particular chemical entry routes depends on several factors, including the intrinsic properties of the pesticide and the biological and ecological characteristics of the particular natural enemy species.

The mirid bug *Nesidiocoris tenuis* (Reuter) (Hemiptera: Miridae) is a generalist zoophytophagous predator of multiple pests that target tomato plants and other agricultural crops [12,13,14]. *N. tenuis* is mass-reared and released for crop protection in enhanced biocontrol programs designed to combat whiteflies and, most recently, the invasive moth *Tuta absoluta* (Meyrick) (Lepidoptera: Gelechiidae) [15,16,17,18]. Additionally, *N. tenuis* also contributes to the control of thrips, mites, aphids, spider mites, leaf miners and some other lepidopteran pests in greenhouse and field environments [19,20,21]. Thus, given the value of *N. tenuis* as a biological control agent, it must become a focus of research on the lethal and sublethal effects of potential IPM pesticides.

The concurrent use of pesticides and alternative control agents is common in IPM programs. However, these combinations are not always compatible: some studies have demonstrated that predatory insects (i.e., natural enemies) can be more susceptible to chemical pesticides than the pests they prey upon [22,23,24]. As potential alternatives to chemical pesticides in IPM, plant derivatives, including naturally occurring compounds such as essential oils, are typically safer for humans and the environment, and they may be more compatible with natural enemies, which themselves play crucial roles by reducing the risk of pest reappearance and the amount of pesticide used [25]. Therefore, the impact of pesticides on natural enemies should always be considered when selecting chemical controls in IPM. Indeed, the use of selective pesticides, which affect insect pests, but are relatively harmless to natural enemies, will conserve natural enemies and thereby contribute to the success of IPM programs [26].

In recent years, attempts have been made to develop pesticides of natural origin that are more environmentally friendly than synthetic pesticides [27]. Benzyl methyl ester, also known as methyl benzoate (MB), is a volatile organic compound (often derived from fermented apple juice) found in many plant species [28]. MB has recently been shown to have acute toxic effects on various insect species including whiteflies, aphids and mites [29,30,31,32], which are the prey of *N. tenuis*. Possible non-target effects of MB on natural enemies have yet to be fully investigated.

Here, the lethal and sublethal effects of MB on the important biological control agent *N. tenuis* were investigated. The ultimate goal of the present study was to enhance our knowledge of pest control methods suitable for IPM programs, and specifically to ensure that MB was safe for use with *N. tenuis*. Our results showed that the insecticidal toxicity of 1% MB could be classified as harmless to *N. tenuis* according to the International Organization for the Biological and Integrated Control of Noxious Animals and Plants, West Palearctic Regional Section.

## 2. Materials and Methods

### 2.1. Insects and Reagents

In 2017, adults of *N. tenuis* were sourced from the Insect Industry Research Institute in Nonsan, Korea. Since 2017, a colony of *N. tenuis* was maintained in a cage (45 × 60 × 90 cm) containing potted tomato plants (*Lycopersicon lycopersicum*) infested with the sweet potato whitefly *Bemisia tabaci* MED (Gennadius) (Hemiptera: Aleyrodidae). Sucrose solution (20%) was provided as a supplementary food source for *N. tenuis*. Rearing conditions were 25 °C ± 1 °C, 60% ± 10% relative humidity (RH) and a 16:8 h L:D photoperiod. Plants were replaced as required.

MB (Cat. M29908-500G), Tween 20 (Cat. P1379-500G) and Tween 80 (Cat. P1754-25ML) were purchased from Sigma-Aldrich (St. Louis, MO, USA). MB solutions (0.25%, 0.5% and 1%) were prepared according to the developed method published by Mostafiz et al. [30]. These MB concentrations were tested on *N. tenuis* because they are known to be suitable for pest control of whiteflies, aphids and mites [30,31,32]. Distilled water containing 0.5% Tween 20 (*v/v*) and 0.5% Tween 80 (*v/v*) was used as a negative control. In the toxicity experiments, acetamiprid (a neonicotinoid insecticide) in the commercial formulation Mospiran^®^ (8%, SC at 10 g 20 L^−1^; Farm Hannong, Seoul, Korea) was used as a positive control due to its demonstrated toxicity to whiteflies [33,34] and hemipteran predators [35].

### 2.2. Lethal Effects of MB on N. tenuis

To evaluate the residual contact toxicity of MB under laboratory conditions, tomato plants grown in a greenhouse were treated with MB using a hand-held sprayer (100-mL volume) until the point of run-off (10 mL/plant). Treatment groups were as follows: 0.25%, 0.5% and 1% MB; the negative control; and the positive control, which was expected to cause high mortality of *N. tenuis*. Leaves with dry residues were collected after 2 h and transferred to the laboratory. The petiole of each leaf was inserted into a water-filled Eppendorf tube (1.5 mL) to maintain leaf turgidity during the experiment; the tube was then placed into a breeding cup (12 cm diameter × 8 cm height). Sucrose solution (20%) was supplied in each breeding cup as a food source for the bugs. Fifteen adults of *N. tenuis* (<3 days old) of both sexes were placed on test leaves in each breeding cup, and the number of surviving and dead adults were recorded daily for 5 days. Each treatment was replicated three times using 15 insects per replication. The adult bugs were considered dead when they remained immobile after being touched with a fine paintbrush. These experiments were carried out under laboratory conditions at 25 °C ± 1 °C, 60% ± 10% RH and with a 16:8 h L:D photoperiod.

To evaluate the residual contact toxicity of MB under greenhouse (43 m^2^) conditions, tomato plants with three leaves (20 leaflets) were sprayed with the three MB solutions (0.25%, 0.5% and 1%), the negative control or the positive control using a hand-held sprayer (10 mL/plant). After spraying, plants were maintained in greenhouse conditions until the residues were completely dried. Each plant was then placed into a mesh-covered cage (50 × 50 × 40 cm). Subsequently, adults of *N. tenuis* (n = 20) were released into these cages. Sucrose solution (20%) was again supplied as a food source for the bugs. Three replications were carried out for each MB concentration and each control. Mortality was recorded daily for 7 days using the process described above. These experiments were carried out at 25 °C ± 1 °C, 40% ± 10% RH and with a 16:8 h L:D photoperiod.

### 2.3. Sublethal Effects of MB on N. tenuis

The antifeedant effect of MB on the adults of *N. tenuis* (<3 days old) was assessed under laboratory conditions using the method developed by Roditakis et al. [2] and Fytrou et al. [33]. First, adults of *B. tabaci* (n = 30) were allowed to lay eggs on fresh tomato plants for 72 h. The number of eggs per leaf was counted under a dissecting microscope (Olympus, Tokyo, Japan). On average, the tomato leaves selected for use in this experiment carried 200 eggs. The test arena was prepared as described in the previous subsection. However, in this experiment, the adults of *N. tenuis* (n = 5) were sprayed directly with the MB solutions (0.25%, 0.5% and 1%), the negative control or the positive control. Each adult of *N. tenuis* was then placed individually on a tomato leaf with the eggs of *B. tabaci*. After adults of *N. tenuis* were placed in the test arena, sublethal antifeedant effects were assessed by counting the number of eggs of *B. tabaci* that each adult of *N. tenuis* consumed daily for 3 days. Three replicates were used for each treatment. These experiments were also carried out at 25 °C ± 1 °C, 60% ± 10% RH and with a 16:8 h L:D photoperiod.

### 2.4. Y-tube Olfactometer Behavioral Assay

**Figure 1:**
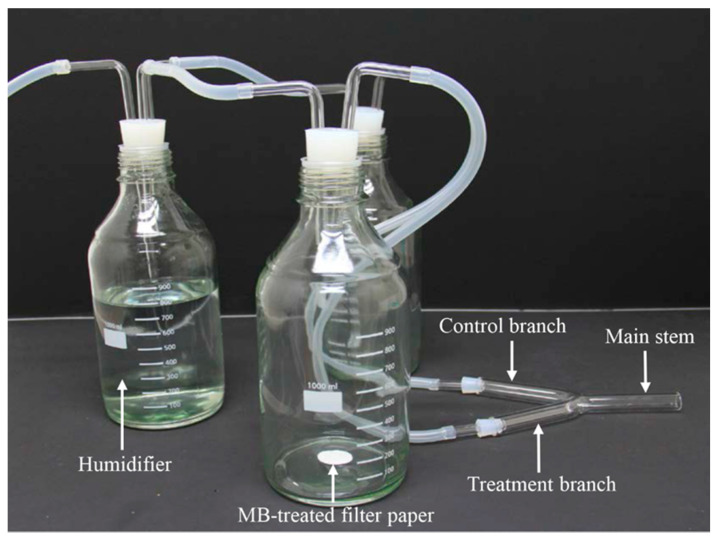
Y-tube olfactometer apparatus used to investigate the olfactory responses of adults of *Nesidiocoris tenuis* to methyl benzoate (MB).

A Y-tube olfactometer, similar to that described by Rahman and Lim [36], was used to analyze the olfactory response of *N. tenuis* to MB. The olfactometer consisted of a Y-shaped glass tube with an inner diameter of 3 cm; the tube had 15-cm-long arms and a 12-cm-long main stem. The angle between the two arms was 45°, whereas the angle between the arm and the main body was 155° (Figure 1). A glass bottle (1 L) with a glass tube (0.9 cm diameter), a Teflon PTEF tube (0.9 cm inner diameter and 1 cm outer diameter), and a cylindrical rubber cork with a hole were connected to each of the arms. A piece of Whatman^®^ filter study was treated with 20 µL of a given MB solution (one of 0.25%, 0.5% or 1% MB), air-dried for 3 min and then introduced into one of the two bottles. The second bottle contained the negative control, which did not contain MB, but instead had filter study with the same volume of distilled water only. Each of these two bottles was also connected by tubes to a third glass bottle filled with distilled water to humidify the flow of compressed artificial air through the test system; this created an air stream of 400 mL/min/arm.

Both male and female adults of *N. tenuis* (5 days old) were starved for 2 h before the olfactory experiments were initiated. They were then individually introduced into the end of the main stem of the olfactometer after the airflow was initiated. When the adult bug traversed more than one-third of the length of either the odor-source (MB treatment) branch or control branch, it was assumed to have made a choice. Insects that remained in the main stem for 10 min were considered to have shown no response. The treatment and control branches were switched after every 10 individuals was tested to minimize directional bias. Data were recorded on the number of adults of *N. tenuis* that approached the MB treatment, the control or that made no choice. Each MB concentration was tested with at least 36 adults as replicates. All experiments were conducted under red light at 25 °C ± 1 °C and 60% ± 10% RH. After each test, the Y-tubes were carefully washed with acetone and distilled water before being allowed to air dry.

### 2.5. Statistical Analysis

One-way ANOVA, followed by a post hoc Tukey’s HSD test, was used to determine differences in toxicity (*P* < 0.05). All percentage mortality data were corrected using Abbott’s formula [37]. Preference data, obtained through the Y-tube olfactometer bioassays, were analyzed using a chi-squared goodness-of-fit test to compare the numbers of insects between the treatment and control branches. The efficiency of MB to reduce the feeding rate of *N. tenuis* was calculated as follows: % reduction = C − T/C × 100, where C is the number of eggs of *B. tabaci* consumed by *N. tenuis* in the control group and T is the number of eggs of *B. tabaci* consumed by *N. tenuis* after a given MB treatment [38]. Proc GLM SAS version 9.4 (SAS Institute, Inc., Cary, NC, USA) was used to conduct all analyses [39]. All graphs were drawn with SigmaPlot 12.5 [40].

Finally, the mortality results were used to classify the pesticide according to the four toxicity categories proposed by the International Organization for Biological Control (IOBC) for laboratory tests: Class 1, harmless (<30% reduction); Class 2, slightly harmful (30%–79% reduction); Class 3, moderately harmful (80%–99% reduction); and Class 4, harmful (>99% reduction) [41,42].

## 3. Results

### 3.1. Lethal Effects of MB on N. tenuis

The results of both experiments (laboratory and greenhouse) assessing the lethal effects of MB on *N. tenuis* are summarized in Table 1. Corrected mortality for *N. tenuis* after 5 days of exposure in the laboratory assay was 17.8%, 8.9% and 4.4% for 1%, 0.5% and 0.25% MB, respectively. In contrast, after 7 days of exposure in the greenhouse, the corrected mortality for *N. tenuis* on MB-treated tomato plants was 13.3%, 6.7% and 3.3% for 1%, 0.5% and 0.25% MB, respectively. Under laboratory and greenhouse conditions, the positive control, acetamiprid, produced the highest corrected mortality of all treatments: 80% after 5 days of exposure in the laboratory test and 66.7% after 7 days of exposure to treated tomato plants in the greenhouse. In both laboratory and greenhouse experiments, the differences between mortality caused by the acetamiprid treatment and mortality caused by the MB concentrations were significant (*df* = 4, 14; laboratory test: *F* = 122.1, *P* < 0.0001; greenhouse test: *F* = 105.8, *P* < 0.0001). According to the IOBC laboratory scale, our results indicate that all three concentrations of MB were harmless (Class 1) to adults of *N. tenuis* after 5 days of exposure, whereas acetamiprid was moderately harmful (Class 2).

### 3.2. Sublethal Effects of MB on N. tenuis

The effects of MB exposure on the feeding rates of *N. tenuis* consuming eggs of *B. tabaci* are summarized in Table 2. The positive control, acetamiprid, produced the greatest antifeedant effect, reducing the feeding rate of *N. tenuis* by 45.4%. In contrast, 1% MB reduced the feeding rate by 22.7%. Indeed, adults of *N. tenuis* exposed to acetamiprid consumed on average 68 eggs in three days, which was significantly lower than the average number of eggs consumed by *N. tenuis* exposed to MB concentrations and the negative control (*df* = 4, 14; *F* = 27.5; *P* < 0.0001) (Table 2). Specifically, adults of *N. tenuis* consumed on average 116, 112 and 96 eggs following exposure to 0.25% 0.5% and 1% MB, respectively. Thus, acetamiprid, but not MB was found to have a significant antifeedant effect on *N. tenuis*.

### 3.3. Olfactory Responses of N. tenuis to MB

**Figure 2:**
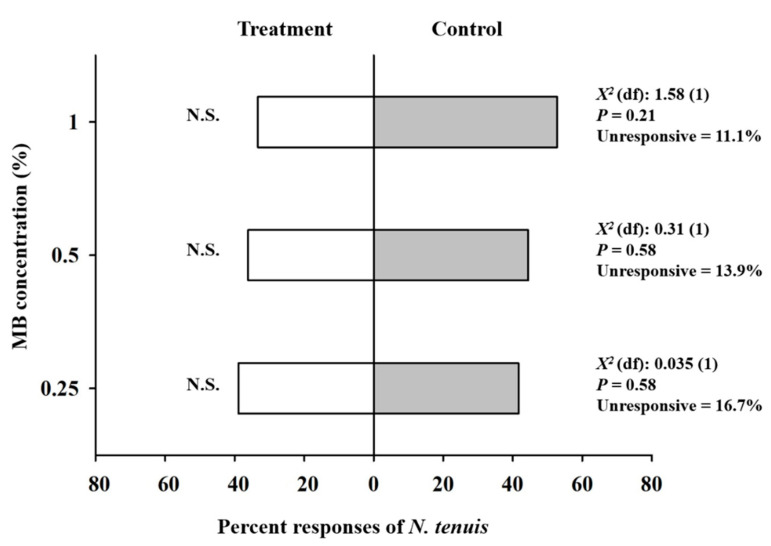
Percent olfactory responses of adults of *Nesidiocoris tenuis* to methyl benzoate (MB) concentrations in a Y-tube olfactometer assay. A response was defined as traversing more than one third of the length of either odor-source branch. N.S. indicates no significant difference between the olfactory responses to an MB concentration and the control (distilled water only).

Overall, in the olfactory experiment, ~80% of the adults of *N. tenuis* made a choice to approach the MB concentration or negative control within the maximum time allowed (10 min). MB concentrations appeared to be somewhat repellent to *N. tenuis*, but there were no significant differences between their repellency and that of the control (Figure 2). Specifically, in the 1% MB test, 33.3% of the predatory bugs chose the 1% MB treatment branch, whereas 52.8% chose the control branch (*χ^2^* = 1.58, *df* = 1, *P* = 0.21). In the other two respective olfactory tests, 36.1% of *N. tenuis* chose the 0.5% MB treatment branch, while 44.4% chose the control branch (*χ^2^* = 0.31, *df* = 1, *P* = 0.58). On the other hand, 38.9% chose the 0.25% MB treatment branch, where 41.7% chose the control branch (*χ^2^* = 0.035, *df* = 1, *P* = 0.85) (Figure 2).

## 4. Discussion

In the present study, exposure to dry residues of MB at 0.25%, 0.5% or 1% on tomato plant tissues did not significantly affect adults of *N. tenuis*. Furthermore, exposures to these MB concentrations through topical spray or foraging on treated plant surfaces did not cause significant mortality or antifeedant effects. Indeed, in contrast to acetamiprid, MB did not reduce the feeding activity (consumption of eggs of *B. tabaci*) of *N. tenuis* which was comparable to that observed in the negative control. Although pesticides can interfere with the feeding behavior of exposed insects due to their repellent or antifeedant properties [1], such effects were not observed for MB. In general, MB exposure resulted in <30% mortality of *N. tenuis*, which corresponds to the harmless category in the IOBC toxicity classification.

MB is highly effective at 1% concentration when used against whitefly, aphid and mite pests: it can cause >70% mortality of these arthropods under laboratory and greenhouse conditions [30,31,32]. In contrast, our present results indicate that 1% MB does not cause high mortality or adverse feeding effects on *N. tenuis*. Differences in mortality between the insect pests and their predators may be due to differential susceptibility to the tested compound or differences in methods of treatment or differences in target stages of the tested organism. We suppose that herbivorous insects are susceptible, but omnivorous insects such as *N. tenuis* are tolerable to MB. Likewise, Campolo et al. [43] recently reported that the essential oil (EO)-based formulations of sweet orange were most toxic against the eggs and larvae of a specialist herbivorous insect pest, *T. absoluta*, whereas least toxic towards the omnivorous predator *N. tenuis*. Further studies are required to find the physiological or biochemical differences between the herbivorous and omnivorous insects after MB treatments.

MB at the concentration that harms whiteflies, aphids and mites can be regarded as relatively harmless to *N. tenuis*. Similarly, Mostafiz et al. [31] reported that the toxicity of 1% MB was very low in a study of the predatory lacewing *Chrysoperla carnea* (Neuroptera: Chrysopidae). Thus, MB would appear to be a strong choice for use as a pesticide in combination with *N. tenuis.* Other pesticides may also work well with *N. tenuis.* Madbouni et al. [44] also reported that the lethal and sublethal effects of pyriproxyfen and spirotetramat on *N. tenuis* were minor (<30% mortality); therefore, these pesticides were considered harmless.

Natural enemies are likely to move between fields, crops and non-crop habitats, creating discontinuous exposure to pesticides. Some pesticides, such as neonicotinoids, not only cause neurotoxic symptoms, but also affect the behavioral parameters of predatory arthropods, e.g., orientation and/or foraging [1]. The present study revealed that MB did not significantly affect the feeding rate of *N. tenuis*. In IPM programs, it is important to consider both the lethal and sublethal effects of a chemical before attempting to use it for crop protection in conjunction with beneficial arthropod populations in order to maximize the natural enemies’ performance against pests. As well as consuming herbivorous insects, *N. tenuis* can feed directly from plant tissues, honeydew and nectar [10,11]. Thus, *N. tenuis* could be exposed to pesticides by feeding on either prey organisms or treated plants. However, we showed that the tested MB concentrations did not harm *N. tenuis* in a sublethal manner. Therefore, our findings indicate that MB is relatively harmless to *N. tenuis* in in vitro assays. Nevertheless, we propose that further testing is required, particularly under field conditions, in order to fully evaluate the effects of MB on these beneficial insects.

## 5. Conclusions

This is the first report on the possible lethal and sublethal effects of MB on the mirid bug *N. tenuis* that is used as a biological control agent. Given that the selection of a suitable insecticide in an IPM program not only depends on its efficacy against the target pest, but also on its toxicity to beneficial insects (as well as its degradation and persistence in the environment), the results of our laboratory and greenhouse experiments support the case for MB as an IPM pesticide. Indeed, we showed that MB is relatively harmless to *N. tenuis*. Future work on the sublethal and residual effects of MB, as well as long-term laboratory and field studies, will further advance our understanding of the impact of this naturally occurring plant toxin on *N. tenuis* and other non-target organisms.

## Figures and Tables

**Table 1:** Corrected cumulative percentage mortality of adults of *Nesidiocoris tenuis* exposed to methyl benzoate (MB) and acetamiprid (positive control) residues on tomato leaves (after 5 days in a laboratory test) or tomato plants (after 7 days in a greenhouse test).

Treatment	Laboratory Condition (Treated Tomato Leaf)	Greenhouse Condition (Treated Tomato Plant)
Acetamiprid	80.0 ± 3.8a	66.7 ± 4.4a
MB 1%	17.8 ± 4.4b	13.3 ± 3.3b
MB 0.5%	8.9 ± 2.2bc	6.7 ± 1.7bc
MB 0.25%	4.4 ± 2.2bc	3.3 ± 1.7bc
Negative control	0.0 ± 0.0c	0.0 ± 0.0c

Data are presented as the mean ± SEM (n = 3). Means within a column followed by the same letters are not significantly different. The negative control comprised distilled water containing 0.5% Tween 20 (*v/v*) and 0.5% Tween 80 (*v/v*).

**Table 2:** Effects of methyl benzoate (MB) on the feeding rates of adults of *Nesidiocoris tenuis* consuming eggs of *Bemisia tabaci.*

Treatment	Mean Number *B. tabaci* Eggs/Treatment	Mean Number of *B. tabaci* Eggs Consumed Per *N. tenuis* Adult/3 Days	Reduction in Feeding Rate Relative to Control (%)
Acetamiprid	212.6 ± 10.1	68.0 ± 5.3c	45.4
MB 1%	208.3 ± 15.5	96.3 ± 4.9b	22.7
MB 0.5%	205.3 ± 6.5	112.3 ± 4.1ab	9.8
MB 0.25%	206.7 ± 13.4	116.0 ± 2.8ab	6.9
Negative control	211.7 ± 8.6	124.6 ± 3.7a	

Data are presented as mean ± SEM (n = 3). Means within a column followed by the same letters are not significantly different. Negative control comprised distilled water containing 0.5% Tween 20 (*v/v*) and 0.5% Tween 80 (*v/v*).
